# “Early transfusion of convalescent plasma in older patients with COVID-19 to prevent disease progression: A structured summary of a study protocol for a randomised controlled trial”

**DOI:** 10.1186/s13063-020-04821-1

**Published:** 2020-10-22

**Authors:** Luciana Teofili, Raffaele Landolfi, Antonella Cingolani, Andrea Antinori, Jacopo Vecchiet, Maurizio Sanguinetti, Antonio Gasbarrini, Tina Pasciuto, Nicoletta Orlando, Silvia Lamonica

**Affiliations:** 1 Fondazione Policlinico Universitario A. Gemelli IRCCS, Rome, Italy; 2 Istituto Nazionale Malattie Infettive Lazzaro Spallanzani IRCCS, Rome, Italy; 3 Presidio Ospedaliero S.S. Annunziata, ASL Lanciano -Vasto-Chieti, Università di Chieti, Chieti, Italy

**Keywords:** COVID-19, randomised controlled trial, protocol, elderly, comorbidities

## Abstract

**Objectives:**

The primary objective is to demonstrate that COVID-19 convalescent plasma (CCP) prevents progression to severe pneumonia in elderly COVID-19 pneumonia patients with chronic comorbidities.

Secondary objectives are to demonstrate that CCP decreases the viral load in nasopharyngeal swabs and increases the anti-SARS-CoV-2 antibody titre in recipients.

**Trial design:**

This is a randomized, open-label, parallel group, phase II/III study with a superiority framework. The trial starts with a screening phase II designed with two-tailed alpha=0.2. In case of positive results, the trial will proceed in a formally comparative phase III (alpha=0.05).

**Participants:**

Adult patients with confirmed or suspected COVID-19 who are at risk according to CDC definition are eligible. Inclusion criteria are all the following: age ≥ 65; pneumonia at CT scan; PaO2/FiO2 ≥300 mmHg; presence of one or more comorbidities; signed informed consent. Exclusion criteria are one of the following: age < 65; PaO2/FiO2 < 300 mmHg; pending cardiopulmonary arrest; refusal to blood product transfusions; severe IgA deficiency; any life-threatening comorbidity or any other medical condition which, in the opinion of the investigator, makes the patient unsuitable for inclusion. The trial is being conducted at three reference COVID-19 centres in the middle of Italy.

**Intervention and comparator:**

Intervention: COVID-19 Convalescent Plasma (CCP) in addition to standard therapy. Patients receive three doses (200 ml/day on 3 consecutive days) of ABO matched CCP.

Comparator: Standard therapy

**Main outcomes:**

A. Primary outcome for Phase II: Proportion of patients without progression in severity of pulmonary disease, defined as worsening of 2 points in the ordinal scale of WHO by day 14.

B. Primary outcome for Phase III: Proportion of patients without progression in severity of pulmonary disease, defined as worsening of 2 points in the ordinal scale of WHO by day 14.

Secondary outcomes for Phase III: Decreased viral load on nasopharyngeal swab at days 6, 9 and 14; Decreased viremia at days 6 and 9; Increased antibody titer against SARS-CoV2 at days 30 and 60; Proportion of patients with negative of SARS-CoV2 nasopharyngeal swab at day 30; Length of hospital stay; Mortality rate at day 28; Total plasma related adverse event (day 60); Total non-plasma related adverse events (day 60); Severe adverse events (SAE) (day 60).

**Randomisation:**

Treatment allocation is randomized with a ratio 1:1 in both phase II and phase III. Randomization sequences will be generated at Fondazione Policlinico Gemelli IRCCS through the RedCap web application. Randomized stratification is performed according to age (under/over 80 years), and sex.

**Blinding (masking):**

None, this is an open-label trial.

**Numbers to be randomised (sample size):**

Phase II: 114 patients (57 per arm).

Phase III: 182 patients (91 per arm)

**Trial Status:**

The trial recruitment started on May 27, 2020. The anticipated date of recruitment completion is April 30, 2021. The protocol version is 2 (May 10, 2020).

**Trial registration:**

The trial has been registered on ClinicalTrials.gov (May 5, 2020). The Identifier number is NCT04374526

**Full protocol:**

The full protocol is attached as an additional file, accessible from the Trials website (Additional file 1). In the interest in expediting dissemination of this material, the familiar formatting has been eliminated; this Letter serves as a summary of the key elements of the full protocol.

**Supplementary information:**

**Supplementary information** accompanies this paper at 10.1186/s13063-020-04821-1.

## Supplementary information

**Additional file 1:.** Full study protocol

## Data Availability

Authors can access data through the REDCap web platform. Data will be available from the authors on reasonable request. Please address the requests to: luciana.teofili@unicatt.it; antonella.cingolani@unicatt.it; raffaele.landolfi@unicatt.it

