# Supplementary material for: “Early transfusion of convalescent plasma in older patients with COVID-19 to prevent disease progression: A structured summary of a study protocol for a randomised controlled trial”
Source: Trials. 2020 Oct 22;21:875. doi: 10.1186/s13063-020-04821-1 (PMC7578576; doi:10.1186/s13063-020-04821-1)

## ADMINISTRATIVE INFORMATION

| | |
|---|---|
| Title | **EARLY TRANSFUSION OF CONVALESCENT PLASMA IN ELDERLY COVID-19 PATIENTS TO PREVENT DISEASE PROGRESSION** |
| Acronymn | **LIFESAVER** (earLy transfusIon oF convalescEnt plaSmA in elderly COVID-19 patients to preVEnt disease pRogression) |
| Trial registration | NCT04374526 |
| Study design | Open label prospective interventional randomized controlled phase II |
| Protocol version | Version 2, May 10,2020 |
| Funding | Not profit study |
| Scientific committee | Raffaele Landolfi; Luciana Teofili; Antonella Cingolani; Andrea Antinori; Gina Zini, Jacopo Vecchiet, Maurizio Sanguinetti; Antonio Gasbarrini |
| Sponsor | Fondazione Policlinico A. Gemelli IRCCS |
| Data management: | Tina Pasciuto and Nicoletta Orlando |
| Study coordinator and monitoring | Silvia Lamonica |
| IDM Committee: | Prof. Gabriella Girelli; Prof. Giuseppe Leone; Prof. Carlo Patrono; Prof. Eligio Pizzigallo. |
| Responsibilities | Scientific committee: study design; management, analysis, and interpretation of data; writing of the report, collection, and management of data. Sponsor: decision to submit the report for publication, and ultimate authority over all the above mentioned activities |
| Participating centers | Fondazione Policlinico A. Gemelli IRCCS (Coordinator center)<br>Istituto Nazionale Malattie Infettive Lazzaro Spallanzani IRCCS<br>Presidio Ospedaliero S.S. Annunziata, ASL Lanciano -Vasto-Chieti |
| **Approval date** | May 13, 2020 |

## STUDY SYNOPSIS

| | |
|---|---|
| **Title** | **Early transfusion of convalescent plasma in elderly COVID-19 patients to prevent disease progression.** |
| **Acronym** | **LIFESAVER** (earLy transfusIon oF convalescEnt plaSmA in elderly COVID-19 patients to preVEnt disease pRogression)) |
| **Participating centers** | Fondazione Policlinico A. Gemelli IRCCS (coordinator center)<br>Istituto Nazionale Malattie Infettive Lazzaro Spallanzani<br>Policlinico SS Annunziata Chieti |
| **Study design** | Open label randomized controlled phase II/III study |
| **Background and rationale** | Older age is an independent poor outcome predictor in hospitalized patients (OR 1.10, 95% CI 1.03–1.17 per year increase). Among 72,314 COVID-19 cases, case fatality rate (CFR) was 2.3% in total population, 8% in people aged 70 to 79, and 14.8% in those aged 80 and older. In the whole population, CFR was higher in people with comorbidities, ranging from 5-6% in persons with hypertension, chronic respiratory disease, diabetes or cancer, up to 10% in those with cardiovascular diseases.<br>Sars-CoV-2 seems to be able to induce a functional exhaustion of specified T and NK lymphocyte subpopulations, breaking down antiviral immunity. One |
| **Hypothesis** | possible explanation is that the immune system of elderly people, might be exhausted by chronic stimulation associated with comorbidities and more susceptible to this Sars-CoV-2 effect. As a result, in these patients, the activation of the innate immune system might fail to produce an adequate adaptive response (i.e., virus-specific CD8+ T-cells). This resulting in persistent self-induced inflammation that eventually causes mortality.<br>We hypothesize that transfusing convalescent plasma (containing neutralizing antibodies) at an early phase of COVID-19 infection could prevent or switch off the persistent inflammatory response elicited by the virus. |
| **Objective** | To demonstrate the superiority of COVID-19 convalescent plasma (CCP) plus standard therapy (ST) over ST alone<br>– To prevent progression of pneumonia in COVID-19 patients aged ≥65 with chronic comorbidities<br>– To decrease viral load<br>– To raise anti-SARS-CoV-2 antibody titer (ELISA, NAT) in recipients |
| **Random 1:1** | **Arm A** (Intervention): COVID-19 Convalescent Plasma (CCP) in addition to standard therapy. Patients receive 3 doses (200 ml/day in 3 consecutive days) of ABO matched CCP.<br>**Arm B** (Control): Standard therapy with no CCP |
| **Phase II endpoints** | **Primary**<br>– Proportion of patients without progression at day 14.<br>**Secondary**<br>– viral load on nasopharyngeal swab at days 6, 9 and 14 |

| | |
|---|---|
| **Phase III endpoints** | **Primary**<br>– Proportion of patients without progression at day 14<br><br>**Secondary**<br>– Viral load on nasopharyngeal swab at days 6,9 and 14<br>– Viremia at days 6 and 9<br>– Antibody titer against SARS-CoV2 at days 30 and 60<br>– Proportion of patients with negative of SARS-CoV2 nasopharyngeal swab at day 30 |
| **Study population** | Adult patients with confirmed COVID-19 |
| **Inclusion criteria** | **Inclusion criteria are all the following:**<br>– Age ≥ 65<br>– evidence of pneumonia by CT scan<br>– PaO2/FiO2 ≥300 mmHg<br>– Presence of one or more comorbidities (list provided in annex 1)<br>– Signed informed consent |
| **Exclusion criteria** | **Exclusion criteria include one of the followings:**<br>– severe COVID-19 disease<br>– PaO2/FiO2 <300 mmHg<br>– impending cardiopulmonary arrest<br>– refusal to blood product transfusions<br>– severe IgA deficiency<br>– any life-threatening comorbidity or any other medical condition which, in the opinion of the investigator, makes the patient unsuitable for inclusion |
| **Sample size** | **Phase II**<br>**114 patients**: 57 per arm<br>increase of patients without progression from 20% to 40%<br>OR 0.375, RR 0.5 (α 0.2 and β 0.2)<br><br>**Phase III**<br>**182 patients**: 91 per arm<br>increase of patients without progression from 20% to 40%<br>OR 0.375, RR 0.5 (α 0.05 and β 0.2) |

## INTRODUCTION

**BACKGROUND.** Blood products collected from convalescent donors have been frequently adopted to provide passive immunization to patients with life threatening diseases. In general, this approach is used when there are no specific vaccines or drugs available for emerging infection-related diseases, namely due to viruses.[1] Different types of blood products may be used to convey passive immunity, including whole blood, plasma, or serum.[2] In addition, immunoglobulins and high-titer immunoglobulins can be obtained from further plasma processing.[2]

**Convalescent plasma in past outbreaks.**

A meta-analysis of Spanish influenza A (H1N1) cases suggested that patients with pneumonia who received influenza-convalescent human blood products seemed to have a reduction in the risk for death.[3] In the modern era, convalescent plasma transfusions were repeatedly administered in several outbreaks with encouraging results, including coronavirus-related Severe Acute Respiratory Syndrome (SARS-CoV) in 2003,[4-6] avian influenza A (H5N1) in 2005,[7,8] and pandemic influenza A (H1N1) in 2009.[9] In the latter condition a multicenter, prospective cohort study, showed that patients receiving convalescent plasma from recovered donors within 5 days of symptom onset had lower viral load and reduced mortality [9] Regarding Ebola, after preliminary positive results, WHO endorsed in 2014 the use of convalescent plasma or serum under blood regulatory authorities.[10,11] The prospective controlled study carried out by the Ebola-Tx Consortium reported that transfusing up to 500 ml of convalescent plasma with unknown levels of neutralizing antibodies in 84 Ebola virus patients was not associated with a significant reduction of mortality.[12] A subsequent study sought to determine whether the level of neutralizing antibodies in different plasma donations could have influenced the response and survival of treated patients.[13] Although more than 90% of donations contained high IgG titers (> 1:1000) at enzyme-linked immunosorbent assay (ELISA), a titer of 1:160 at neutralizing test was found in only 5% of donations.[13] Therefore, in this study, neither viral load or mortality were associated with the dose of neutralizing antibodies received.[13] Positive results in two critically ill

patients affected from Middle East respiratory syndrome coronavirus (MERS-CoV) were related to the presence of high neutralizing antibody titers in the donor plasma.[14] A recent meta-analysis gathering data from 32 studies, suggested that the administration of convalescent plasma or serum is safe and able to reduce mortality in SARS of viral etiology (SARS-CoV, H1N1 and H5N1).[15]

**Experiences in COVID-19 pandemia.**

Three studies have so far reported the use of convalescent plasma in critical COVID-19 patients.[16,17] The first case-series included 5 patients (1 with pre-existing cardiovascular comorbidities) in mechanical ventilation, with a median interval between admission and plasma transfusion of 20 days. All patients had previously received antiviral drugs and methylprednisolone. The titer of neutralizing anti SARS-CoV-2 in donated plasma ranged from 1:80 to 1:480 and the titer of specific anti-SARS-CoV-2 antibodies (anti receptor binding domain IgG and IgM) was higher than 1:1000. Each patient received 400 ml of fresh plasma from a single donor. All treated patients were viremic at the time of plasma infusion and the viral load declined thereafter, along with the clinical recovery.[16] The second study reports 4 cases of COVID-19 in critically ill patients. Three of them had comorbidities (cardiovascular disease, chronic broncopneumopathy, chronic renal failure), and one patient was pregnant. All 4 patients were intubated, received anti-infectious agents for documented bacterial and/or fungal coinfections, and had been treated with additional therapies before plasma infusion (antivirals including interferon, immunoglobulins and/or methylprednisolone). The convalescent plasma was given at variable doses (from 200 ml to 2400 ml). In all patients a progressive decline of viremia was documented after plasma infusion.[17] The third study is under review and is unpublished so far (it is available at the MedRxiv website, a free online archive for complete unpublished and not peer reviewed manuscripts in the medical, clinical, and related health sciences).[18] It is a feasibility pilot study including 10 patients with severe COVID-19 receiving one dose of 200 mL convalescent plasma derived from recently recovered donors (neutralizing antibody titers above 1:640) in addition to maximal supportive care and antiviral agents. Four out of 10 had cardiovascular diseases. The median time from onset of illness to transfusion was 16.5

days. The plasmas were selected among those from 40 donors on the basis of neutralizing titer and ABO compatibility and were subjected to methylene blue photochemistry inactivation for any residual virus. Primary endpoint was the safety of convalescent plasma transfusion and secondary endpoint was the improvement of clinical symptoms and laboratory parameters within 3 days after plasma transfusion. Clinical symptoms significantly improved along with the increase of oxyhemoglobin saturation within 3 days. Several parameters tended to improve as compared to pre-transfusion, including increased lymphocyte counts and decreased C-reactive protein. The viral load was undetectable after transfusion in seven patients with previous viremia, whereas almost all patients in this study had high titers of their own neutralizing antibodies even before plasma infusion.[18] Severe adverse effects due to plasma infusion were not reported in any of these studies.[16-18] Altogether, considering that in all patients plasma infusion was started after several lines of therapies, including anti-infectious agents for concurrent bacterial and fungal infections, the true impact of convalescent plasma on the recovery from COVID-19 cannot be clearly determined.[16-19]

**Convalescent plasma: regulatory issues.**

On March 20, the United States Food and Drug Administration (FDA) approved the use of convalescent plasma as single-patient emergency Investigational New Drug (eIND), a procedure adopted for drug with acceptable safety profile in case of urgent and life threatening conditions with no satisfactory available therapeutic alternatives.[20] The American Association of Blood Banks (AABB) launched a new website as an educational resource to inform the public, blood collectors and clinicians about COVID-19 convalescent plasma (CCP). The aim was to inform and motivate people who had recovered from COVID-19 about donation of convalescent plasma to help improving the status of critically ill patients with COVID-19.[21] FDA indicated that convalescent plasma had to contain an adequate level of SARS-CoV-2 neutralizing antibody titer (e.g., greater than 1:80). Nevertheless, if neutralizing antibody titer could not be determined before donation , FDA invited blood bank to store a retention sample from the convalescent plasma donation for determining antibody titers at a later date.[22] In the meanwhile, AABB released a

protocol to assist facilities in the collection of CCP.[23] American Association of Hematology (ASH) released an information on the same topic, reporting that individual physicians had begun to treat patients with serious COVID-19 disease with convalescent plasma collected by blood centers (according an eIND procedure). In the meanwhile, multicenter clinical trials, focusing on either prophylaxis of high-risk individuals, treatment of those with serious but not critical COVID-19 disease, or treatment of critically-ill ICU patients, were designed.[24] The ASH information clearly reports that use of plasmapheresis to collect large volumes of plasma would be desirable, allowing to treat at least 2-3 recipients by one collection. In addition, quantitative serologic assays are not yet widely available to identify convalescent patients with high titer neutralizing antibodies.[24] Until the time of writing, no official documents have been published on the CCP by the Italian National Blood Centre (Centro Nazionale Sangue (CNS). Nevertheless, current CNS recommendation for blood donor selection during the COVID-19 pandemic, allows individuals with recent COVID-19 infection to donate after 14 days from symptoms cessation and therapy discontinuation.[25] The Italian Society for Apheresis (SIDEM) and the Italian Society of Transfusion Medicine (SIMTI) released a joint position statement endorsing the collection of “hyperimmune convalescent plasma” by Italian blood banks.[26] Hyperimmune convalescent plasma or equivalent definitions are not mentioned in the last version of the “Guide to the preparation, use and quality assurance of blood components”.[27] In the SIDEM/SIMTI document, hyperimmune plasma is defined as the plasma product containing an antibody titer against SARS-CoV-2 higher than 160 at enzyme immunosorbent assay or equivalent tests.[26] This definition was drawn by a Saudi Arabian interventional single group study (ClinicalTrials.gov Identifier: NCT02190799) on convalescent plasma therapy in critical MERS-CoV patients. The study started in 2014 and was withdrawn in 2018. While to our knowledge no results have been published, investigators published the study protocol in which measurement of anti-MERS-CoV antibodies level in donor and participant serum through commercially available kits is recomended.[28] At the time of writing, no commercially available methods to measure anti SARS-CoV2 antibodies are available, either based on ELISA or neutralization

assays. Moreover, the equivalence between presence of an adequate titer of neutralizing antibody (NAT) and ELISA detectable antibodies is not straightforward. For example, studies on Ebola virus infection showed that 90% or more among convalescent plasma donors exhibited high IgG and IgM levels at ELISA, whereas only 5% of them had high NAT.[13] Conversely, among 881 convalescents from H1N1 pandemic influenza, a high NAT (> 1:40) was found in more than 90% of individuals and was associated with more severe disease, with higher levels in patients experiencing pneumonia in comparison with those with afebrile presentation (OR 3.39; 95% CI 1.49-7.61).[29] Similar findings were reported in a small number of patients with MERS-CoV infection, with patients with pneumonia showing long lasting antibody titers >40.[30] Therefore, donor selection should rely on the anamnestic basis, in order to identify donors potentially exhibiting high NAT. Finally, it should be emphasized that the majority of studies reporting a positive effect of convalescent plasma against SARS-CoV, H1N1 and H5N1 do not report the NAT of transfused plasma.[15] Indeed, whereas it is conceivable that convalescent plasma donors might exhibit variable levels of NAT or ELISA-dosed antibodies, an evidence-based higher efficacy of "hyperimmune" plasma (as defined by the SIDEM/SIMTI document)[26] is still lacking.

**RATIONALE**

Our hypothesis is that transfusing COVID-19 convalescent plasma (CCP) to elderly patients with COVID-19 pneumonia and comorbidities might prevent the evolution to severe disease. At present, no single specific vaccine, nor specific antiviral therapy against COVID-19 can be used to modify the clinical course of this disease. COVID-19 can present with different severity of symptoms and can be categorized as mild, severe, or critical COVID-19. Mild COVID-19 includes non-pneumonia and mild pneumonia cases (with no need for oxygen supplementation). Severe COVID-19 is characterized by dyspnea, respiratory frequency ≥30/minute, blood oxygen saturation ≤93%, PaO2/FiO2 ratio <300, and/or lung infiltrates increasing >50% within 24–48 hours). Critical COVID-19 includes respiratory failure, septic shock, and/or multiple organ dysfunction/failure, including acute kidney injury and cardiac injury.[31] An important lesson learned from initial reports on hospitalized patients is

that COVID-19 rapidly deteriorate in specific categories of patients.[32-34] Poor prognosis patients are aged over 60, are mainly man and near half of them have some comorbidities.[34] In-hospital death is associated with older age, higher Sequential Organ Failure Assessment (SOFA) score and blood d-dimer greater than 1 µg/mL. These findings are known to be associated with severe pneumonia.[31] Although current best estimate is that about 81% of people with COVID-19 have mild disease and never require hospitalization, 14% of patients have severe illness and need oxygen therapy, and approximately 5% require intensive care treatment and mechanical ventilation.[31] Since the reported case fatality rate ranges from 49% to 97% in critical patients, it is pivotal to find alternative strategies to prevent older patients and patients with chronic morbidities from worsening.[31-34] The association between severe COViD-19 and specified patient' characteristics has emerged from the analysis of large patient population[31] and from meta-analysis of recently published studies.[35] Older age is an independent poor outcome predictor in hospitalized patients (odds ratio 1.10, 95% CI 1.03–1.17 per year increase).[34] Among 72,314 COVID-19 cases, case fatality rate was 2.3% in total population, 8% in people aged 70 to 79, and 14.8% in those aged 80 and older.[31] In the whole population, case-fatality rate was higher in people with comorbidities, ranging from 5-6% in persons with hypertension, chronic respiratory disease, diabetes or cancer, up to 10% in those with cardiovascular diseases.[31] On the other side, one or more comorbidities was reported in 36/54 (67%) poor outcome COVID-19 patients as compared to 55/137 (40%) survivors (p= 0·0010).[34] These overall data show that elderly people with comorbidities are most susceptible to severe COVID-19 and therefore, should be protected by approaches which may supplement current therapies. Data relative to the long lasting persistence of the virus in patients with poor outcome, together with the presence of lymphopenia (CD4+T and CD8+ T cells) and exaggerated pro-inflammatory and anti-inflammatory cytokines (IL-2R, IL-6,TNF-α and IL-10) suggest that disease severity and outcome of COVID-19 is due to the SARS-CoV-2-induced cytokine storm.[36] Sars-CoV-2 seems able to induce a functional exhaustion of specified T and NK lymphocyte subpopulations, breaking down antiviral immunity.[37] One possible explanation is that the immune system of elderly people,

exposed to chronic stimulation associated with comorbidities, might be more susceptible to this Sars-CoV-2 effect.[37] As a result, in these patients, the activation of the innate immune system might fail to produce an adequate adaptive response (i.e., virus-specific CD8+ T-cells).[38] This could result in a persistent self-induced inflammation that then causes mortality.[38] We therefore hypothesize that transfusing the convalescent plasma containing neutralizing antibodies at an early phase of disease could prevent or switch-off the persistent inflammatory response elicited by the virus, before that cytokine storm exerts devastating effects. As a proof of concept that CCP has an activity on the infection, in enrolled patients we will assess the viral load in blood and nasopharyngeal swab before and plasma infusion.

**REFERENCES**


1. Keller MA, Stiehm ER. Passive immunity in prevention and treatment of infectious diseases. Clin Microbiol Rev 2000; 13: 602-14.
2. Marano G, Vaglio S, Pupella S, et al. Convalescent plasma: new evidence for an old therapeutic tool? Blood Transfus. 2016;14:152-157.
3. Luke TC, Kilbane EM, Jackson JL, et al. Meta-analysis: convalescent blood products for Spanish influenza pneumonia: a future H5N1 treatment? Ann Intern Med 2006; 145:599–609.
   *Metanalysis of 8 studies, 1703 treated patients*
4. Soo YO, Cheng Y, Wong R, et al. Retrospective comparison of convalescent plasma with continuing high-dose methylprednisolone treatment in SARS patients. Clin Microbiol Infect. 2004l;10:676-8
   *Retrospective analysis of 40 patients: 19 received high-dose methylprednisolone followed by convalescent plasma and 21 received only high-dose methylprednisolone. Convalescent plasma was associated with shorter hospital stay and lower mortality. No immediate adverse effects are reported.*
5. Yeh KM, Chiueh TS, Siu LK, et al. Experience of using convalescent plasma for severe acute respiratory syndrome among healthcare workers in a Taiwan hospital. J Antimicrob Chemother. 2005;56(5):919-22.
   *Case series; 10 patients.*

6. Cheng Y, Wong R, Soo YO, et al. Use of convalescent plasma therapy in SARS patients in Hong Kong. Eur J Clin Microbiol Infect Dis 2005; 24:44-6

*Retrospective cohort (80 patients)*

7. Kong LK, Zhou BP. Successful treatment of avian influenza with convalescent plasma. Hong Kong Med J. 2006;12(6):489.

*Case report; one patient.*

8. Zhou B, Zhong N, Guan Y. Treatment with convalescent plasma for influenza A (H5N1) infection. N Engl J Med. 2007;357(14):1450-1.

*Case report; one patient.*

9. Hung IF, To KK, Lee CK, et al. Convalescent plasma treatment reduced mortality in patients with severe pandemic influenza A (H1N1) 2009 virus infection. Clin Infect Dis. 2011;52(4):447 56.

*Prospective cohort study. Twenty patients versus 73 controls (all admitted to ICU): mortality in plasma-group 20.0% vs 54.8%; mortality OR in plasma-group 0.20; 95% CI 0.06-0.69*

10. Mupapa K, Massamba M, Kibadi K, et al. Treatment of Ebola hemorrhagic fever with blood transfusions from convalescent patients. J Infect Dis 1999; 179: Suppl 1: S18-23.

11. WHO. (2014) Use of convalescent whole blood or plasma collected from patients recovered from Ebola virus disease for transfusion, as an empirical treatment during outbreaks. Available at http://apps.who.int/iris/rest/bitstreams/604045/retrieve *Last accessed March 31,2020*

12. van Griensven J, Edwards T, de Lamballerie X, et al; Ebola-Tx Consortium. Evaluation of Convalescent Plasma for Ebola Virus Disease in Guinea. N Engl J Med. 2016;374(1):33-42.

13. van Griensven J, Gallian P, de Lamballerie X. Convalescent Plasma and the Dose of Ebola Virus Antibodies. N Engl J Med. 2017;376(13):1297.

14. Ko JH, Seok H, Cho SY, et al. Challenges of convalescent plasma infusion therapy in Middle East respiratory coronavirus infection: a single centre experience. Antivir Ther. 2018;23(7):617-622.

15. Mair-Jenkins J, Saavedra-Campos M, Baillie JK, et al; Convalescent Plasma Study Group. The effectiveness of convalescent plasma and hyperimmune immunoglobulin for the treatment of severe acute

respiratory infections of viral etiology: a systematic review and exploratory meta-analysis. J Infect Dis. 2015;211:80-90.
16. Shen C, Wang Z, Zhao F, Yang Y, et al. Treatment of 5 Critically Ill Patients With COVID-19 With Convalescent Plasma. JAMA. 2020 Mar 27 [Epub ahead of print]
17. Zhang B, Liu S, Tan T, et al. Treatment with convalescent plasma for critically ill patients with SARS-CoV-2 infection. Chest. 2020 Mar 31. [Epub ahead of print]
18. https://www.medrxiv.org/content/10.1101/2020.03.16.20036145v1 (last accessed April 9,2020)
19. Chen L, Xiong J, Bao L, Shi Y. Convalescent plasma as a potential therapy for COVID-19. Lancet Infect Dis. 2020;20:398-400.
20. https://www.fda.gov/media/136470/download (last accessed April 2)
21. http://www.aabb.org/press/Pages/pr200403.aspx (last accessed April 5)
22. https://www.fda.gov/vaccines-blood-biologics/investigational-new-drug-ind-or-device-exemption-ide-process-cber/investigational-covid-19-convalescent-plasma-emergency-inds
23. http://www.aabb.org/advocacy/regulatorygovernment/Documents/COVID-19-Convalescent-Plasma-Collection.pdf (last accessed April 4)
24. https://www.hematology.org/covid-19/covid-19-and-convalescent-plasma
25. (*https://www.centronazionalesangue.it/node/836*).
26. http://www.emaferesi.it/wp-content/uploads/2020/03/Convalescent-Plasma-SIMTI_SIDEM-1.pdf (last accessed April 3,2020)
27. European Directorate for the Quality of Medicines & HealthCare. Guide to the preparation, use and quality assurance of blood components. Recommendation No. R (95) 15. 19th Edition (2017)
28. Arabi Y, Balkhy H, Hajeer AH, et al. Feasibility, safety, clinical, and laboratory effects of convalescent plasma therapy for patients with Middle East respiratory syndrome coronavirus infection: a study protocol. Springerplus. 2015 Nov 19;4:709.
29. Hung IF, To KK, Lee CK, et al. Effect of clinical and virological parameters on the level of neutralizing antibody against pandemic influenza A virus H1N1 2009. Clin Infect Dis. 2010;51:274-9.

30. Choe PG, Perera RAPM, Park WB, et al. MERS-CoV Antibody Responses 1 Year after Symptom Onset, South Korea, 2015. Emerg Infect Dis. 2017; 23:1079-1084.
31. Wu Z, McGoogan JM. Characteristics of and Important Lessons From the Coronavirus Disease 2019 (COVID-19) Outbreak in China: Summary of a Report of 72 314 Cases From the Chinese Center for Disease Control and Prevention. JAMA. 2020 Feb 24. doi: 10.1001/jama.2020.2648. [Epub ahead of print]
32. Chen N, Zhou M, Dong X, et al. Epidemiological and clinical characteristics of 99 cases of 2019 novel coronavirus pneumonia in Wuhan, China: a descriptive study. Lancet 2020.
33. Wang D, Hu B, Hu C, Zhu F, Liu X, Zhang J et al. Clinical characteristics of 138 hospitalized patients with 2019 novel coronavirus-infected pneumonia in Wuhan, China. JAMA. 2020. Epub 2020/02/08. doi: 10.1001/jama.2020.1585
34. Zhou F, Yu T, Du R, et al. Clinical course and risk factors for mortality of adult inpatients with COVID-19 in Wuhan, China: a retrospective cohort study. Lancet 2020. published online March 9. https://doi.org/10.1016/ S0140-6736(20)30566-3.
35. Emami A, Javanmardi F, Pirbonyeh N, Akbari A. Prevalence of Underlying Diseases in Hospitalized Patients with COVID-19: a Systematic Review and Meta-Analysis. Arch Acad Emerg Med. 2020 Mar 24;8(1):e35. eCollection 2020. Review
36. Chen G, Wu D, Guo W, Cao Y, Huang D, Wang H, et al. Clinical and Immunologic features in severe and moderate Coronavirus Disease 2019. J Clin Invest. 2020.
37. Zheng M, Gao Y, Wang G, Song G, Liu S, Sun D, Xu Y, Tian Z. Functional exhaustion of antiviral lymphocytes in COVID-19 patients. Cell Mol Immunol. 2020 Mar 19. doi: 10.1038/s41423-020-0402-2. [Epub ahead of print]
38. Ahmadpoor P, Rostaing L. Why the immune system fails to mount an adaptive immune response to a Covid -19 infection. Transpl Int. 2020 Apr 1. doi:10.1111/tri.13611. [Epub ahead of print]

## BENEFIT / RISK ASSESSMENT OF THE TREATMENT

Benefit of CCP. Potential benefit of CCP is to prevent progression of COVID-19 to a more severe form. Benefit of plasma infusion are conceivably connected with the presence of specific antibodies against the virus. At the time of writing, there are no validated commercially available tests to determine the NAT against SARS-CoV2 in collected units. Nevertheless, as soon as methods for NAT or ELISA determination will be available, CCP will be selected on the basis of the antibody titre in order to select units with higher activity.

Risks of CCP. Potential risks connected with the use of CCP are those relative to general blood transfusion and include allergic/febrile reaction (estimated frequency 1 in 100), transmission of infectious disease (HIV, HBV, HCV, syphilis, etc.; estimated frequency 1 in 1.000.000), transfusion associated circulatory overload (TACO, estimated frequency 1 in 100) and transfusion associated acute lung injury (TRALI, estimated frequency 1 in 100.000). Hemolytic non severe reactions could also occur if ABO incompatible plasma is transfused. Any precaution will be undertaken to minimize these risks. Transfused CCP will fulfil all current quality and safety requirements for blood products will be collected according to CNS recommendations for blood donor selection during the COVID-19 pandemic. All units will be subjected to pathogen inactivation by Intercept technology. To prevent TRALI, female donors who had pregnancies or donors who received transfusions will be not allowed to donate. An additional theoretical risk is that antibody anti-SARS-CoV-2 might prevent patients from their active immunization. For this reason, the trial will test patients for serology for SARS-CoV-2, and if negative, they should be vaccinated when anti-SARS-CoV-2 will be available. Finally, a phenomenon named “Antibody Dependent Enhancement” (ADE) of infection has been observed in in vitro studies, infecting cells with different types of viruses in presence of specific antibodies. Theoretically, ADE might worsen infection in COVID-19 patients. It is unknown, however, if ADE might occur in vivo in COVID-19 patients and several clinical studies have reported that SARS-CoV-specific antibodies found in SARS patients are not harmful in this regard.

## OBJECTIVES AND OVERALL STUDY DESIGN

| | |
|---|---|
| **Objectives** | The primary objective of this study is to demonstrate that COVID-19 convalescent plasma (CCP) may prevent progression to severe pneumonia in elderly COVID-19 pneumonia patients with chronic comorbidities. |
| **Trial design** | This is a randomized phase II/III study designed to evaluate the efficacy of convalescent plasma, when used in combination with standard therapy to prevent the progression of pneumonia in elderly COVID-19 patients with comorbidities. The trial will start with a screening Phase II trial designed with two-tailed alpha=0,2. In case of positive result the trial will proceed in a formally comparative phase III trial (alpha=0.05) Three studies have so far reported the use of CCP in ventilated patients without recording adverse events, but data on patients in earlier phase of disease are lacking. For this reason, the first 57 patients will be observed in order to assess preliminarily the safety of CCP in this population. |
| **Study setting** | Participating centers are:<br>– Fondazione Policlinico Gemelli IRCCS (coordinating center)<br>– Istituto Nazionale Malattie Infettive Lazzaro Spallanzani IRCCS<br>– Ospedale SS Annunziata, Chieti |

## ELIGIBILITY CRITERIA

| | |
|---|---|
| **Study population** | Adult patients with confirmed or suspected COVID-19 who are at risk according to CDC definition are eligible for the study. |
| **Inclusion criteria** | **All the following**<br>– Age $\geq 65$<br>– pneumonia at CT scan<br>– PaO2/FiO2 $\geq$300 mmHg<br>– Presence of one or more comorbidities (consider the list provided in Appendix A)<br>– Signed informed consent |
| **Exclusion criteria** | **One of the following**<br>– Age < 65 |

- PaO2/FiO2 < 300 mmHg
- pending cardiopulmonary arrest
- refusal to blood product transfusions
- severe IgA deficiency
- any life-threatening comorbidity or any other medical condition which, in the opinion of the investigator, makes the patient unsuitable for inclusion.

**INTERVENTIONS**

**Treatment** Patients will be randomized 1: 1 to receive one of the following treatments

- **Arm A (Intervention):** Standard therapy with COVID-19 Convalescent Plasma (CCP). Patients in this arm receive three consecutive doses (200 ml/day for 3 days) of ABO matched CCP.
- **Arm B. (Control):** Standard therapy with no CCP

**Stopping rule** The study will be discontinued if 6 (10%) trial participants in the arm A will have a severe adverse event connected with plasma infusion (Appendix B).

**Concomitant therapies** Patients enrolled in this study receive standard therapies in addition to CCP. The enrollment of participants in other interventional trials is not allowed.

**OUTCOMES AND ENDPOINTS**

**Phase II** **Primary outcome**

- Proportion of patients without progression in severity of pulmonary disease defined as worsening of 2 points in the ordinal scale of WHO within day 14.

The WHO ordinal scale to assess the disease progression is provided in Appendix C.

**Secondary outcome**

- Decreased viral load on nasopharyngeal swab (days 6 and 9)

**Phase III** Phase III of the study will start soon after completing the analysis of data relative to phase II and if the primary outcome of phase II is met.

**Primary outcome**

- Proportion of patients without progression in severity of pulmonary disease defined as worsening of 2 points in the ordinal scale of WHO within day 14.

**Secondary outcomes**

- Decreased viral load on nasopharyngeal swab (days 6, 9 and 14)
- Decreased viremia at day 6 and 9
- Increased antibody titre against SARS-CoV2 (days 30 and 60)
- Proportion of patients with negative of SARS-CoV2 nasopharyngeal swab (day 60)
- Length of hospital stay
- Mortality rate at day 28
- Total plasma related adverse event (Febrile reactions, TRALI, TACO, TTI) (day 60)
- Total non-plasma related adverse events (day 60)
- Severe adverse events (SAE) (day 60)

Appendix C provides the definitions for adverse events and severe adverse events.

**Timeline** The study timeline is shown in the Gantt diagram (Appendix D)

**METHODS: STUDY ASSESSMENT AND PROCEDURES**

**Procedures and visits** A study flow-chart is shown in Appendix E.

**Screening phase: day -1**

**Baseline visit: day 0:**

Physical examination and vital signs (blood pressure, heart rate, respiratory rate, temperature), Respiratory status (pO2/FiO2 and SpO2 at rest if applicable), ECG; blood sampling for ABO groups (on two separate samples), RT-PCR for SARS-CoV-2 on nasopharyngeal swab. routine blood tests (blood count, creatinine, BUN, sodium, potassium, ALT, AST, LDH, fibrinogen, D-dimer, ferritin, CRP), viremia, anti SARS-Cov-2 antibody screening, IL6.

**Plasma administration**: **days 1,2, 3.** All patients will sign the informed consent to receive blood products in use at each center.

**Daily study assessment: days 1 to 14**

Physical examination and vital signs (blood pressure, heart rate, respiratory rate, temperature, oxygen supplementation), Respiratory status ($pO_2/FiO_2$ and $SpO_2$ at rest if applicable), ECG and chest scan (on day 7 and 14, or additional days if clinically advised); routine blood tests (blood count, creatinine, BUN, sodium, potassium, ALT, AST, LDH, fibrinogen, D-dimers, ferritin, CRP, every three days unless clinically advised), RT-PCR SARS-Cov2 on rhino-pharyngeal swab at the same time points of routine blood tests until 2 consecutive negative swabs within 24 hours, viremia (day 6, day 9, day 12 and day 14), anti SARS-Cov-2 antibody screening (day 14), IL6 (day 3, day 6, day 9).

**Day 15 visit (or visit at discharge)**

Physical examination and vital signs (blood pressure, heart rate, respiratory rate, temperature)

**Day 30 visit**

physical examination and vital signs and data on respiratory status and oxygen supplementation, anti SARS-Cov-2 antibody screening

**Day 60 visit**

physical examination and vital signs and data on respiratory status and oxygen supplementation, anti SARS-Cov-2 antibody screening.

**Sample size**

**Phase II.** To demonstrate that adding CCP to standard therapy is associated with an increase of proportion of patients without progression from 20% (control, Arm B) to 40% (treatment, Arm A), with a 80% power and a two-tailed alpha error of 0.2, 114 (57 per arm) evaluable patients are needed, with an OR for treatment of 0.375.

**Phase III.** To demonstrate that adding CCP to standard therapy is associated with a reduction of the proportion of patients progressing to severe pneumonia from 40% (control, Arm B) to 20% (treatment, Arm A), with a 80% power and a two-tailed alpha error of 0.05, 182 (91 per arm) evaluable patients are needed, with an OR for treatment of 0.375.

Chow, SC, Shao J and Wang H. 2003. Sample Size Calculations in Clinical Research. Marcel Dekker. New York.

**Recruitment**

Patients are recruited at three different COVID centers (Fondazione Policlinico Gemelli IRCCS, Istituto Nazionale Malattie Infettive Lazzaro Spallanzani and Policlinico SS Annunziata Chieti). Convalescent plasma

will be collected at Fondazione Policlinico Gemelli IRCCS, Azienda Ospedaliera San Camillo Forlanini and Ospedale SS Annunziata Chieti.

## METHODS: ASSIGNMENT OF INTERVENTIONS

**Allocation and sequence generation**

Treatment allocation will be randomized between arms A and B with a ratio 1:1 and will be the same in phase II and phase III. Randomization sequences will be generated at Fondazione Policlinico Gemelli IRCCS.R through the RedCap web application, uploading the allocation table through the randomization module (ref: https://www.project-redcap.org/).

Randomized stratification will performed according to age (under/over 80 years), and sex.

**Blinding (masking)**

The assignment to intervention will be unmasked to all trial participants (care providers, outcome assessors, and data analysts)

## METHODS: DATA COLLECTION, MANAGEMENT, AND ANALYSIS

**Data collection methods**

A customized eCRF (eletronic Case Report Foarm) will be created for the study. Study data will be collected and managed using REDCap electronic data capture tools hosted at Fondazione Policlinico Universitario A. Gemellli, IRCCS (https://redcap-irccs.policlinicogemelli.it/). REDCap (Research Electronic Data Capture) is a secure, web-based application designed to support data capture for research studies, providing 1) an intuitive interface for validated data entry; 2) audit trails for tracking data manipulation and export procedures; 3) automated export procedures for seamless data downloads to common statistical packages; and 4) procedures for importing data from external sources [Ref1: PA Harris, R Taylor, R Thielke, J Payne, N Gonzalez, JG. Conde, Research electronic data capture (REDCap) – A metadata-driven methodology and workflow process for providing translational research informatics support, J Biomed Inform. 2009 Apr;42(2):377-81. Ref 2: PA Harris, R Taylor, BL Minor, V Elliott, M Fernandez, L O'Neal, L McLeod, G Delacqua, F Delacqua, J Kirby, SN Duda, REDCap Consortium, The REDCap consortium: Building an international community of software partners, J Biomed Inform. 2019 May 9 [doi: 10.1016/j.jbi.2019.103208].

**Data management** Each participating site must maintain appropriate medical and research records for this trial and regulatory/institutional requirements for the protection of confidentiality of study subjects. The Principal Investigator is responsible for assuring that the data collected are complete, accurate, and recorded in a timely manner.

**Statistical methods** **Primary endpoint:**

Based on the above-mentioned design, the comparison of proportion of progression free patients will be made between A vs B through the Fisher exact test at the completion of phase II and phase III.

**Secondary endopoints**

Descriptive results will be presented as means ± standard deviation (SD), medians with interquartile range (IQR), and percentages with 95% confidence intervals (CI). Chi-square or Fisher's exact test will be used to compare categorical variables. ANOVA and Student's t-test will be used for continuous variables unless they will be not normally distributed, in which case the Kruskal-Wallis or Mann-Whitney test will be used to compare continuous parameters. Kaplan-Meier product-limit estimates will be used to calculate time to different secondary endpoints. The log-rank test will be used to assess the difference between the survival curves among arms. Changes in some biomarkers respect to baseline values will be compared using paired Wilcoxon test. The association between primary outcome and transfusion dose of CCP expressed as ml/kg of the recipient body weight, adjusted for anti-Sars-CoV-2 neutralizing and ELISA antibody concentrations, will be investigated through regression analysis. All tests will be two-sided and a P value inferior to 0.05 will be regarded as significant.

**Analysis settings** Analysis will be carried out in the "intention to treat" set.

**METHODS: MONITORING**

**Data monitoring** Each study site agrees to allow monitors from Monitoring Unit of Fondazione Policlinico Gemelli (FPG) IRCCS direct access to the study records and medical records from those patients enrolled in the clinical study. In accordance with the applicable regulations and good clinical practice (GCP), the monitor shall periodically contact the center. The

duration, nature and frequency of such visits/contacts shall depend on the rate of recruitment, the quality of the documents in the possession of the center, and its adherence to the protocol.

Through these contacts, the monitor must: control and evaluate the progress of the study, examine the collected data, conduct Source Document Verification (SDV), identify every problem and find solutions.

The aims of the monitoring activity are to verify that: the rights and well-being of the subject are respected, the study data are accurate, complete and verifiable by original documents and the study is conducted in accordance with the protocol and any approved amendments, GCP and the applicable regulations.

## ETHICS AND DISSEMINATION

**Research ethics approval**

The study will be conducted with the approval of the Ethics Committee, after verification of compliance with the European Union Clinical Practice Standards and in accordance with ICH Good Clinical Practice (GCP) and the ethical principles expressed in Declaration of Helsinki. The study will be carried out adhering to local legal requirements and the applicable national law, whichever represents the greater protection for the individual. Study protocol, patient information and informed consent will be submitted to the appropriate Ethical Committee for approval. Will inform the Ethical Committee about any changes in the study protocol which could interfere with the patient's safety.

**Protocol amendments**

Any protocol amendments will be communicated (e.g., changes to eligibility criteria, outcomes, analyses) to investigators, EC/IRBs, trial participants, trial registries, and regulators.

**Informed consent for patients**

The participant, adequately informed in clear, simple and understandable words of the technical terms used, will be invited to provide written informed consent. The participant will be provided with a description of the general aims of the research, the methodology and procedures used, the indication of any benefits or possible risks and adverse effects. In addition to the consent to participate to the study, all patients will sign the consent to receive blood products in use at each center. The physicians treating the hospitalized patient are responsible for information of the patient and obtaining of the Informed Consent. The consent will be

expressed orally: two different witnesses will state that the patient was properly informed and fully understood study aim and procedures, and will sign the consent. In the event that the interested party revokes consent to the processing of data for research purposes, the biological sample taken for such purposes would also be destroyed. Blood samples will be collected during routinely performed samples during the investigations necessary for the pathology in progress. Participation in the research will not entail any additional costs for the participant. Should a medical problem arise due to the study, the participant will be provided with the most appropriate treatment. In accordance with the law on the protection of personal data (Legislative Decree 30/6/2003 No. 196, Guidelines for the processing of personal data in the context of clinical trials of medicinal products - 24 July 2008 - OJ No. 190 of August 14, 2008; 2016/679 European Regulation, as well as the Deliberation of the Guarantor (Del.52 of 24/7/08) will be specified that the centers of experimentation in accordance with the responsibilities established by the rules of good clinical practice (legislative decree 211/2003), will process personal data, especially those on health and, only to the extent that they are indispensable in relation to the objective of the study, other data related to the demographic characteristics, exclusively according to the realization of the study.

The informed consent for patients is provided in Appendix F.

**Informed consent for donors**

An informed consent, asking for their availability to be contacted by the medical staff of transfusion centers, will be provided to patients not enrolled in this study who are discharged from hospital after COVID-19. These patients will be informed about the possibility to donate their plasma and modalities of plasma donation. If they agree to participate to the study, they will be contacted after discharge and plasma donation will be arranged if their donor suitability will be confirmed.

The informed consent for patients is provided in Appendix G.

**Confidentiality**

All subject related information including Case Report Forms, laboratory specimens, evaluation forms, reports, etc. will be kept strictly confidential. All records will be kept in a secure, locked location and only research staff will have access to the records. Subjects will be identified only by means of a coded number specific to each subject. All computerized databases

will identify subjects by numeric codes only, and will be password protected.
Upon request, subject records will be made available to the study audit, monitoring representatives of the study promoter, or representatives of regulatory agencies (CNS).

**Access to data** Only people officially registered as study investigators or data managers will receive a user login to access the REDCap web platform and enter/manage data. Source documentation should support the data collected on the CRF's. Source documents include all recordings of observations or notations of clinical activities and all reports and records necessary for the evaluation and reconstruction of the clinical

**Insurance** After study approval by EC, the sponsor will stipulate an insurance policy with Lloyd's Insurance Company S.A to cover all risks connected with plasma therapy.

**Dissemination policy** Investigators and sponsor will communicate trial results to participants, healthcare professionals, and the public through scientific publications. Authorship eligibility will be defined according to ICMJE guidelines.

**APPENDICES**

**APPENDIX A** **LIST OF COMORBIDITIES**

a. Chronic cardiac disease
b. Hypertension
c. Metastatic solid tumors
d. Chronic pulmonary disease
e. Malignant neoplasm (including leukemia & lymphoma)
f. AIDS/HIV
g. Chronic kidney disease requiring dialysis
h. Obesity (BMI≥40)
i. Liver disease
j. Diabetes (with or without complications)
k. Rheumatologic disorder
l. Chronic neurological disorder
m. Hemiplegia
n. Paraplegia

**APPENDIX B** **ADVERSE/SEVERE ADVERSE EVENT: DEFINITIONS AND MANAGEMENT**

**Adverse Events** An ‘adverse event’ is defined in Article 2 (m) of Directive 2001/20/EC as follows: ‘Any untoward medical occurrence in a patient or clinical trial subject administered a medicinal product and which does not necessarily have a causal relationship with this treatment’. An adverse event can therefore be any unfavorable and unintended sign (including an abnormal laboratory finding, for example), symptom or disease temporally associated with the use of a medicinal product, whether or not considered related to the medicinal product

An “adverse event” is any worsening in the general condition of a subject, or a subject participating in a clinical experiment to whom a pharmaceutical product is administered, regardless of its relationship with the given treatment. An AE may also be any unexpected adverse sign (which can include an abnormal laboratory result of clinical significance), and each symptom or pathology temporarily associated with the use of a pharmaceutical product, regardless of its relationship with the same product. AEs may be **expected** (consistent with the information leaflet provided with the product) or **unexpected** (inconsistent with the information available).

<u>Adverse events include:</u>

• The exacerbation of a pre-existing pathology;

• An increase in the frequency or intensity of an episodic event or pre-existing condition;

• A condition occurring or diagnosed after the administration of the study drug, even if current before the start of the study;

• Persistent diseases/symptoms at the baseline visit that worsen after the start of the study.

<u>Adverse events do NOT include:</u>

• Medical or surgical procedures (e.g. surgery, endoscopy, tooth extraction, transfusions), but the condition requiring the procedure is an adverse event

• Diseases or conditions present at the beginning of the study that have not worsened, but remained stable during the course of the study.

• Situations in which no unexpected adverse event has occurred (e.g. hospital admission for elective cosmetic surgery/social problems).

• An overdose of antiviral agents or concomitant drugs without onset of symptoms or associated signs.

• Laboratory abnormalities deemed by the investigator to be of no clinical significance.

Adverse events will be graded according with CTCAE v5.0. (Appendix B) Grade 1 and Grade 2 events are not considered adverse events, but details of these events must be documented in detail in the subject's study files.

Stable chronic conditions which are present prior to clinical trial entry and do not worsen are not considered adverse events and will be accounted for in the subject's medical history.

**Serious Adverse Events *(SAE's*).** A 'serious adverse event' is defined in Article 2(o) of Directive 2001/20/EC as follows: 'Any untoward medical occurrence or effect that at any dose results in death, is life-threatening, requires hospitalization or prolongation of existing hospitalizations, results in persistent or significant disability or incapacity, or is a congenital anomaly or birth defect'. These characteristics/consequences have to be considered at the time of the event. For example, regarding a life-threatening event, this refers to an event in which the subject was at risk of death at the time of the event; it does not refer to an event which hypothetically might have caused death if it were more severe. Some medical events may jeopardize the subject or may require an intervention to prevent one of the above characteristics/consequences. Such events (hereinafter referred to as 'important medical events') should also be considered as 'serious' in accordance with the definition. Medical and scientific judgement should be exercised in deciding whether an event is 'serious' in accordance with these criteria

A Serious Adverse Event is defined as an SAE meeting one of the following:

• Death during the period of protocol-defined surveillance

• Life Threatening Event (defined as a participant at immediate risk of death at the time of the event)

• In-patient hospitalization or prolongation of existing hospitalization during the period of protocol-defined surveillance

• Results in congenital anomaly or birth defect
• Results in a persistent or significant disability/incapacity

Any other important medical event that may not result in one of the above outcomes, may be considered a serious adverse experience when, based upon appropriate medical judgment, the event may jeopardize the participant and may require medical or surgical intervention to prevent one of the outcomes listed above.

**AE/SAE relationship assignment.** For all collected AE's/SAE's, the clinician who examines and evaluates the subject will determine the adverse event's causality based on temporal relationship and his/her clinical judgment. The degree of certainty about causality will be graded using the categories below:

• Definitely Related: There is clear evidence to suggest a causal relationship, and other possible contributing factors can be ruled out. The clinical event, including an abnormal laboratory test result, occurs in a plausible time relationship to drug administration and cannot be explained by concurrent disease or other drugs or chemicals. The response to withdrawal of the drug (de-challenge) should be clinically plausible. The event must be pharmacologically or phenomenologically definitive, with use of a satisfactory rechallenge procedure if necessary.

• <u>Probably Related</u>: There is evidence to suggest a causal relationship, and the influence of other factors is unlikely. The clinical event, including an abnormal laboratory test result, occurs within a reasonable time sequence to administration of the drug, is unlikely to be attributed to concurrent disease or other drugs or chemicals, and follows a clinically reasonable response on withdrawal (de-challenge). Re-challenge information is not required to fulfill this definition.

• <u>Possibly Related</u>: There is some evidence to suggest a causal relationship (e.g., the event occurred within a reasonable time after administration of the trial medication). However, the influence of other factors may have contributed to the event (e.g., the subject's clinical condition, other concomitant events). Although an adverse drug event may rate only as "possibly related" soon after discovery, it can be flagged as requiring more information and later be upgraded to "probably related" or "definitely related", as appropriate.

• Unlikely: A clinical event, including an abnormal laboratory test result, whose temporal relationship to drug administration makes a causal relationship improbable (e.g., the event did not occur within a reasonable time after administration of the trial medication) and in which other drugs or chemicals or underlying disease provides plausible explanations (e.g., the subject's clinical condition, other concomitant treatments).

• Not related: The AE is completely independent of study drug administration, and/or evidence exists that the event is definitely related to another etiology. There must be an alternative, definitive etiology documented by the clinician.

**SAE reporting procedures.** The information to be reported for each SAE is: the date of event occurrence, the date of event resolution, a brief description of the event, concomitant therapy, lab test reports, maximum intensity of the event or correlation with the agent. If there is any change in the information over time, an updated SAE report must be sent. All adverse reactions simultaneously defined as serious and unexpected must be reported by the investigator to the study sponsor as soon as possible.

The investigator will report all SAE to Eudravigilance through the specific form, to Ethical Committees, and to the manufacturer, within the timelines of the article 17 of the European Directive 2001/20/EC. The investigator will provide an annual Development Safety Update Report, including all Serious Adverse Events occurring in the Study, to the Regulatory Agency, and to the Ethical Committee as per local requirements.

Notification deadlines:

• if, in addition to being serious and unexpected, a SAE is also fatal or life-threatening, a preliminary SAE report must be completed as soon as possible and, in any case, within 24 hours after being informed about the event.

• For all other serious and unexpected adverse events, the investigator must complete a SAE report as soon as possible after the manifestation of the event and, in any case, no later than 15 days after becoming aware of the event.

**Monitoring of AE'S/SAE'S.** Any AE that occurs between the times a study participant signs the informed consent form and the time s/he departs the study at the end of the final follow-up visit (or at the time of

early discontinuation of the subject from the study for any reason) will be captured and recorded. At each contact with the subject, the investigator (or designate) must seek information on adverse events by specific questioning and, as appropriate, by examination.

All AEs and SAEs must be followed up:

• until their complete resolution

• until their stabilization

• until the event can be attributed a new etiology

• until the patient ceases to be in the care of the Centre

The investigator must ensure that the follow-up reports include all supplementary information allowing a complete evaluation of the nature and/or the cause-effect relationship of the AE or SAE, including further laboratory and other tests, pathology reports, and any specialist examinations.

**Suspected unexpected serious adverse reactions (SUSARS).** The investigator shall ensure that all relevant information about suspected serious unexpected adverse reactions that are fatal or life-threatening is recorded and reported as soon as possible to the Database Eudravigilance, and to the Ethics Committee, and in any case no later than seven days after knowledge by the sponsor of such a case, and that relevant follow-up information is subsequently communicated within an additional eight days.

All other suspected serious unexpected adverse reactions shall be reported to the database Eudravigilance concerned and to the Ethics Committee concerned as soon as possible but within a maximum of 15 days of first knowledge by the sponsor. The sponsor shall also inform all investigators.

<u>Adverse reaction</u> — causality An adverse reaction' is defined in Article 2(n) of Directive 2001/20/EC as follows: 'all untoward and unintended responses to an investigational medicinal product related to any dose administered'. The definition covers also medication errors and uses outside what is foreseen in the protocol, including misuse and abuse of the product. The definition implies a reasonable possibility of a causal relationship between the event and the IMP. This means that there are facts (evidence) or arguments to suggest a causal relationship.

Unexpected’ adverse reaction - Definition: Article 2(p) of Directive 2001/20/EC defines ‘unexpected adverse reaction’ as follows: ‘an adverse reaction, the nature or severity of which is not consistent with the applicable product information (e.g. investigator’s brochure for an unauthorised investigational product or summary of product characteristics for an authorised product)’.

The term ‘severity’ is used here to describe the intensity of a specific event. This has to be distinguished from the term ‘serious’. Reports which add significant information on the specificity, increase of occurrence, or severity of a known, already documented serious adverse reaction constitute unexpected events

## APPENDIX C WHO ORDINAL SCALE FOR ASSESSING PROGRESSION OF PNEUMONIA[1]

1 = Death;

2 = Hospitalized, on invasive mechanical ventilation or extracorporeal membrane oxygenation (ECMO);

3 =Hospitalized, on non-invasive ventilation or high flow oxygen devices;

4 = Hospitalized, requiring supplemental oxygen;

5 = Hospitalized, not requiring supplemental oxygen;

6 = Not hospitalized

1. Coronavirus disease (Covid-19) R&D. Geneva: World Health Organization https://www.who.int/blueprint/priority-diseases/key-action/novel-coronavirus/en/

## APPENDIX D

## Gantt diagram

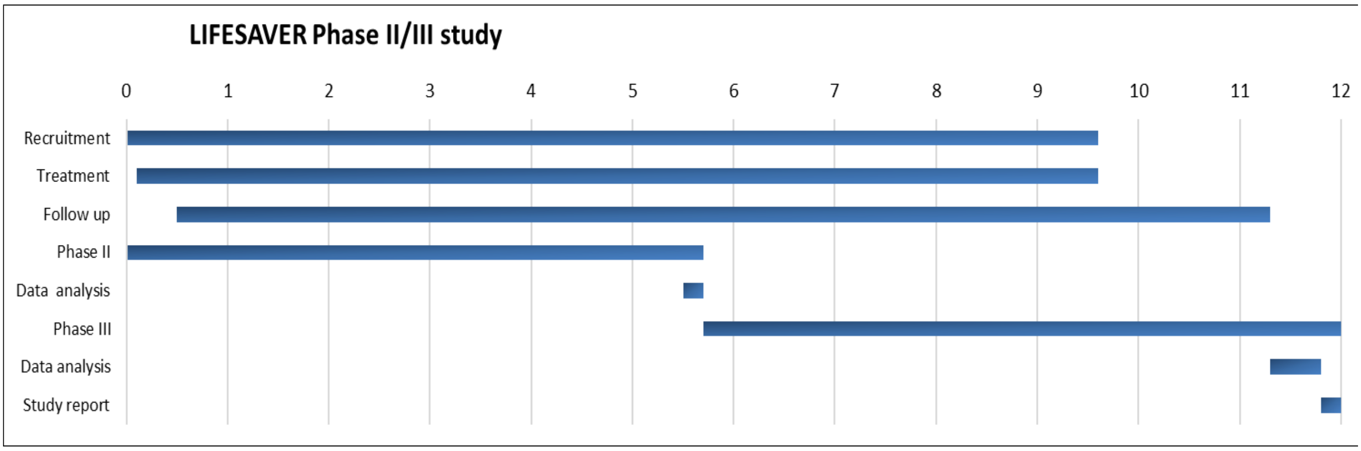

## APPENDIX E.

# LIFESAVER – Study flow chart

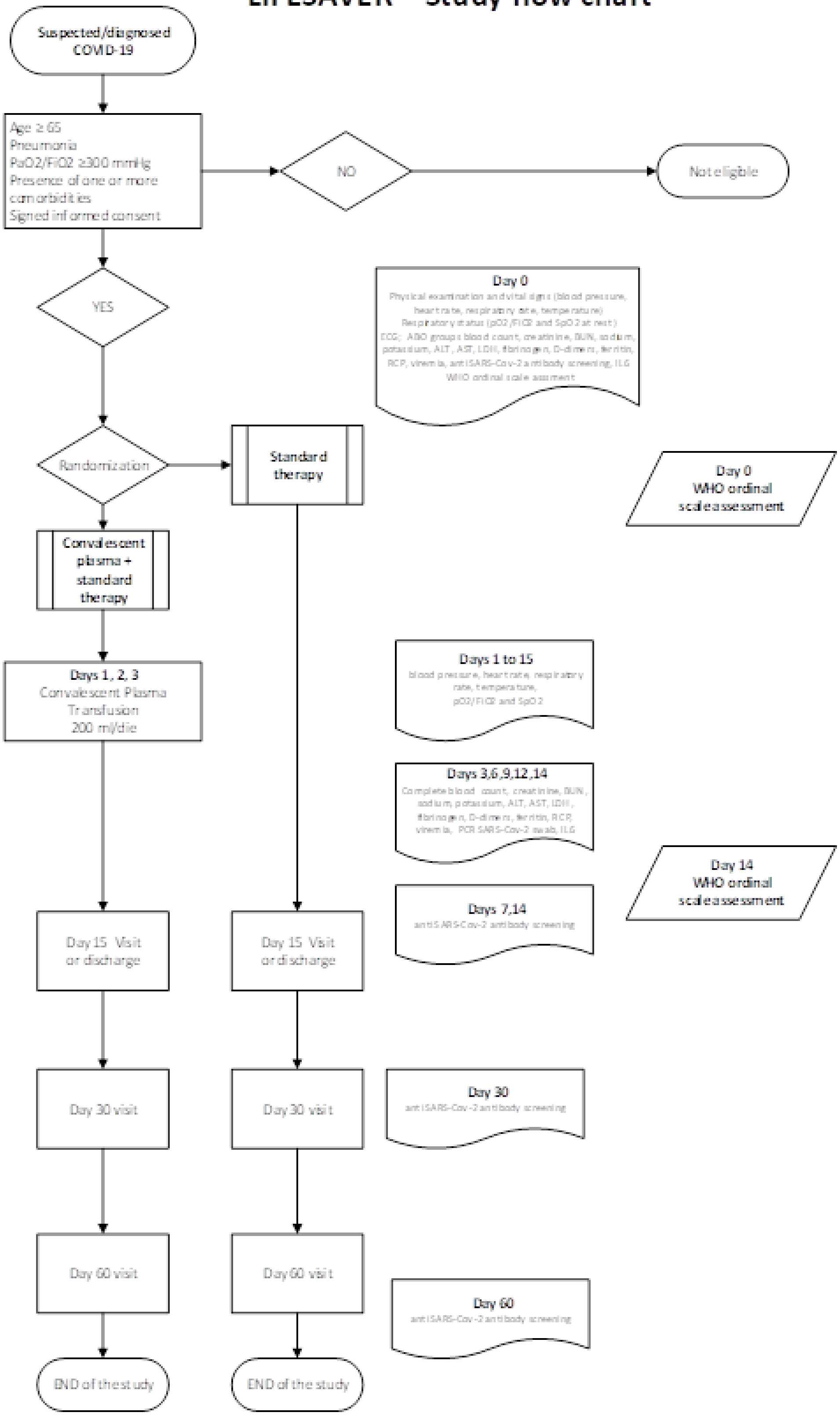

Blood Bank : Convalescent Plasma collection flow chart

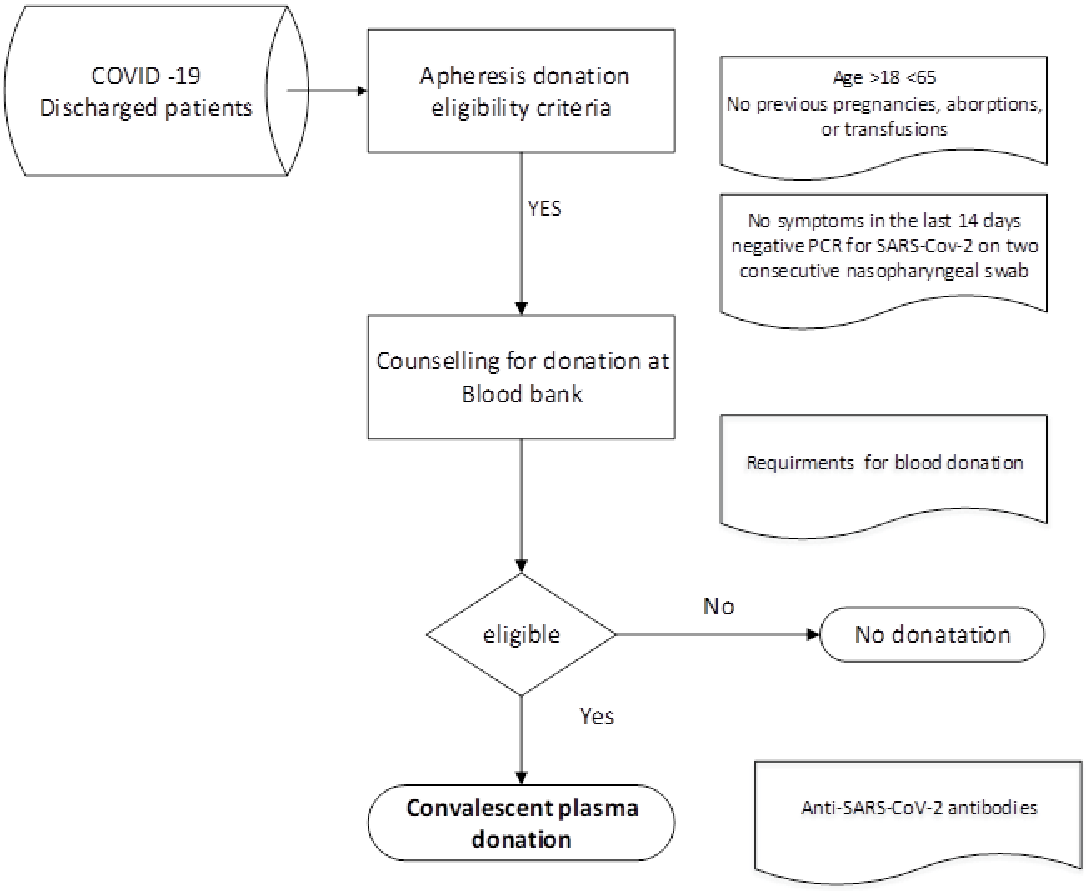

Supplement: Supplementary file 1 — Additional file 1:. Full study protocol [file 13063_2020_4821_MOESM1_ESM.pdf]
